# COVID-19 Artificial Intelligence Diagnosis Using Only Cough Recordings

**DOI:** 10.1109/OJEMB.2020.3026928

**Published:** 2020-09-29

**Authors:** Jordi Laguarta, Ferran Hueto, Brian Subirana

**Affiliations:** MIT AutoID Laboratory 2167 Cambridge MA 02139 USA; MIT AutoID Laboratory 2167 Cambridge MA 02139 USA; Harvard University 1812 Cambridge MA 02138 USA

**Keywords:** AI diagnostics, convolutional neural networks, COVID-19 screening, deep learning, speech recognition

## Abstract

*Goal:* We hypothesized that COVID-19 subjects, especially including asymptomatics, could be accurately discriminated only from a forced-cough cell phone recording using Artificial Intelligence. To train our MIT Open Voice model we built a data collection pipeline of COVID-19 cough recordings through our website (opensigma.mit.edu) between April and May 2020 and created the largest audio COVID-19 cough balanced dataset reported to date with 5,320 subjects. *Methods:* We developed an AI speech processing framework that leverages acoustic biomarker feature extractors to pre-screen for COVID-19 from cough recordings, and provide a personalized patient saliency map to longitudinally monitor patients in real-time, non-invasively, and at essentially zero variable cost. Cough recordings are transformed with Mel Frequency Cepstral Coefficient and inputted into a Convolutional Neural Network (CNN) based architecture made up of one Poisson biomarker layer and 3 pre-trained ResNet50's in parallel, outputting a binary pre-screening diagnostic. Our CNN-based models have been trained on 4256 subjects and tested on the remaining 1064 subjects of our dataset. Transfer learning was used to learn biomarker features on larger datasets, previously successfully tested in our Lab on Alzheimer's, which significantly improves the COVID-19 discrimination accuracy of our architecture. ***Results:* When validated with subjects diagnosed using an official test, the model achieves COVID-19 sensitivity of 98.5% with a specificity of 94.2% (AUC: 0.97). For asymptomatic subjects it achieves sensitivity of 100% with a specificity of 83.2%**. *Conclusions:* AI techniques can produce a free, non-invasive, real-time, any-time, instantly distributable, large-scale COVID-19 asymptomatic screening tool to augment current approaches in containing the spread of COVID-19. Practical use cases could be for daily screening of students, workers, and public as schools, jobs, and transport reopen, or for pool testing to quickly alert of outbreaks in groups. General speech biomarkers may exist that cover several disease categories, as we demonstrated using the same ones for COVID-19 and Alzheimer's.

## I. Introduction

Strict social measures in combination with existing tests and consequently dramatic economic costs, have proven sufficient to significantly reduce pandemic numbers, but not to the extent of extinguishing the virus. In fact, across the world, outbreaks are threatening a second wave, which in the Spanish flu was way more damaging than the first one [1]. These outbreaks are very hard to contain with current testing approaches unless region-wide confinement measures are sustained. This is partly because of the limitations of current viral and serology tests and the lack of complementary pre-screening methods to efficiently select who should be tested. They are expensive making the cost of testing a whole country each day impossible, e.g. $8.6B for the US population alone assuming a $23 test [2]. And to be effective, they often require subjects remain isolated for a few days until the result is obtained. In contrast, our AI pre-screening tool could test the whole world on a daily, or even hourly basis at essentially no cost. In terms of capacity, in the week leading up to July 13, 2020, daily diagnostic testing capacity in the United States was fluctuating between 520,000 and 823,000 tests. However, certain experts forecasted the need for 5 million tests per day by June, increasing to 20 million tests per day by July [3]. The unlimited throughput and real-time diagnostic of our tool could help intelligently prioritize who should be tested, especially when applied to asymptomatic patients. In terms of accuracy, in an evaluation of nine commercially available COVID-19 serology tests, in early phase (7-13 days after onset of disease symptoms) sensitivities vary between 40-86% and AUC vary between 0.88-0.97 [4]. Meanwhile, our tool with AUC 0.97 achieves 98.5% sensitivity.

It has been proposed optimal region-wide daily testing and contact tracing could be a close substitute to region-wide confinement in terms of stopping the spread of the virus [5] and avoid the costs of stopping the economy. However, many current attempts at testing, contact tracing, and isolation like the UK initially employed, have been far from successful [6]. This is mainly caused by many countries lacking the tools at the time to employ an agile, responsive, and affordable coordinated public health strategy [6]. Therefore, as the virus spreads to countries who cannot afford country-wide daily testing nor confinement, a large-scale, low-cost, and accurate pre-screening tool may be essential to prioritize tests for rapidly detecting and locally preventing outbreaks. Different AI approaches have recently been proposed to support the management of the pandemic [7]–[10].

An AI coughing test would provide some advantage that may partially offset the issues with existing biological testing approaches. Capabilities of our AI tests include: non-invasive real-time results, essentially zero variable cost, accessible by anyone, and capable to longitudinally monitor patients.

As any AI deep learning approach, we needed training data and a modelling strategy. To address the data component, which was not available, we initiated a worldwide crowd-sourced effort to collect COVID-19 forced-cough audios along with 10 multiple choice questions related to the diagnosis of the disease and relevant symptoms as shown in Table I. Our MIT Open Voice COVID-19 Cough dataset [10] sets a new benchmark as the largest audio health dataset with several hundred thousand coughs of which 5,320 COVID-19 positive and negative balanced subjects were selected for this research. We selected all COVID-19 positives and, randomly, an equivalent number of negative ones from the rest of our dataset.

**TABLE I:** The Selection for the COVID-19 Subjects for Performance Comparison Aimed to Reproduce a Scenario Where Subjects Are Requested to Voluntarily Use a Screening Tool. That Is Why the Ratio Is Not Exactly Balanced in Terms of Any Specific Demographic Statistic. Instead, We Chose the Split to Reflect the Voluntary Participation in Our Crowd-Sourcing Exercise, Which in the Case of COVID-19 Positives Was 41.8% Male, 53.0% Female and 8.9% Other Because That Was the Ratio of Voluntary Participants. Note the Ratio of Control Patients Included a 6.2% More Females, Possibly Eliciting the Fact That Male Subjects Are Less Likely to Volunteer When Positive. Thus, the Percentages Reflect Our Sample and Therefore Produce What We Feel Is the Best Estimate of Overall Performance if a Screening Tool Was Voluntarily Used at Scale. In Any Case, Our Extensive Database Allows Selective Training for Other Demographics. Note the ‘Hit’ Column Shows the Model Accuracy on Each Respective Subgroup. The Categories *Personal, Doctor* and *Official* Correspond to the Source of Diagnostic Entered by Each Subject, Whether They Took an Official Test, Had a Doctor's Diagnosis, or Simply a Personal Assessment. The Last Row Shows the Results of Applying the Same Biomarkers to Alzheimer's [11]

	**Positives**			**Negatives**			**Total**	
	**#**	**%**	**Hit(%)**	**#**	**%**	**Hit(%)**	**%**	**Hit(%)**
Number of Patients	2660	50.0	94.0	2660	50.0	78.4	100.0	86.4
**COVID-19 Diagnostic**								
Official Test	475	17.9	98.5	224	8.4	94.2	13.1	97.1
Doctor Assessment	962	36.2	98.8	523	19.7	92.8	27.9	96.7
Personal Assessment	1223	46.0	89.5	1913	71.9	72.6	58.9	79.2
**Symptoms**								
No Symptoms 'Official'	102	3.8	100.0	114	4.3	83.2	4.1	91.1
No Symptoms	196	7.4	100.0	2029	76.3	78.3	41.8	80.2
Fever	656	24.7	98.1	34	1.3	88	13.0	97.6
Tiredness	1428	53.7	93.2	210	7.9	81.2	30.8	91.7
Sore Throat	1064	40.0	99.9	205	7.7	84.8	23.9	97.5
Diff. Breathing	680	25.6	99.3	49	1.8	77.1	13.7	97.8
Chest Pain	680	25.6	99.4	58	2.2	87.3	13.9	98.4
Diarrhea	652	24.5	94.2	100	3.8	71.8	14.1	91.2
Cough	1724	64.8	99.8	262	9.8	91.1	37.3	98.7
**Gender**								
Male	1116	42.0	94.4	1263	47.4	78.9	44.7	86.2
Female	1308	49.2	94.6	1212	45.6	78.1	47.4	86.7
Other	236	8.9	93.8	185	7.0	77.2	7.9	86.5
**Alzheimer's Diagnostic**								
Doctor Diagnostic	78	100.0	100.0	78	50.0	87.5	100.0	93.8

To address the modelling strategy, we were inspired by our research on Alzheimer's [11] and the growing evidence of recently reported symptoms of COVID-19 patients who suffered neurological impairments such as temporary neuromuscular impairment and loss of smell during and post infection [12]–[14].

After trying unsuccessfully a few basic CNN models, the connection between COVID-19 and the brain is what led us to pivot the COVID-19 modelling efforts to our Open Voice Brain Model framework (OVBM), based on the Brain Model of the MIT Center for Brain Minds and Machine [15], since we had recently applied it to the diagnostic of Alzheimer's achieving above state-of-the-art accuracy of 93.8%. Our MIT OVBM framework is based on orthogonal acoustic biomarkers to diagnose and create an individualized patient saliency map to longitudinally monitor patients [11].

In the following sections we present the data collection pipeline for this study (Section II.A), an overview of our COVID19 AI model (Section II.B), the four biomarkers (Section II.C) and the results (Section III), including our model performance on pre-screening COVID-19 subjects, followed by an evaluation of the biomarkers and our individualized patient longitudinal saliency map. We conclude in Sections IV and V with a brief summary, limitations, and implications on suggested next steps for the deployment in practice of our COVID-19 pre-screening tool, suggesting a pooling strategy, and, more broadly, implications of our approach for the role of AI in Medicine going forward.

## II. Methods

### A. COVID-19 Cough Dataset

Approved by the MIT COHUES Institutional Review Board, in April 2020 we initiated a worldwide cough data collection effort of through our website recording engine (opensigma.mit.edu) with the aim of creating the MIT Open Voice dataset for COVID-19 cough discrimination [10]. We collected variable length cough audio recordings (on average 3 coughs per subject) accompanied by a set of 10 multiple choice questions related to the diagnosis of the disease and general subject information: age, sex, country, region; whether, when and outcome of medical diagnosis done and whether the source of diagnosis was an official test, a doctor's evaluation or a personal assessment; and finally information about symptoms and days since their onset. Symptoms requested included fever, tiredness, sore throat, difficulty breathing, persistent pain or pressure in the chest, diarrhoea and coughing.

So far, we have an estimated subject count of 2,660 COVID-19 positives and a 1-10 ratio of positive to control subjects. Recording was available on various browsers and devices, reducing any possible device specific bias. Data was anonymized before being collected on our secure server and samples were saved without compression in WAV format (16kbs bit-rate, single channel, opus codec). Samples that had no audio content (e.g. where the file was 44 bytes) were removed. No segmentation was performed on the cough recordings used to train and test.

We used all the COVID-19 positive samples in our dataset and randomly selected the same number of COVID-19 negative subjects for a balanced distribution. We only used samples with two conditions, first a diagnostic had been done in the last 7 days and, second, with symptoms onset no longer than 20 days and where symptoms continued until the sample was captured. The subject forced-cough audios and diagnostic results were used to train and validate the COVID-19 discriminator. 4256 subjects (80%) were used for training and 1064 (20%) for validation. Table I provides more details on the patient distribution for the randomly sampled patients selected from the dataset.

### B. Overview of the COVID-19 Model Architecture

**Fig. 1.**
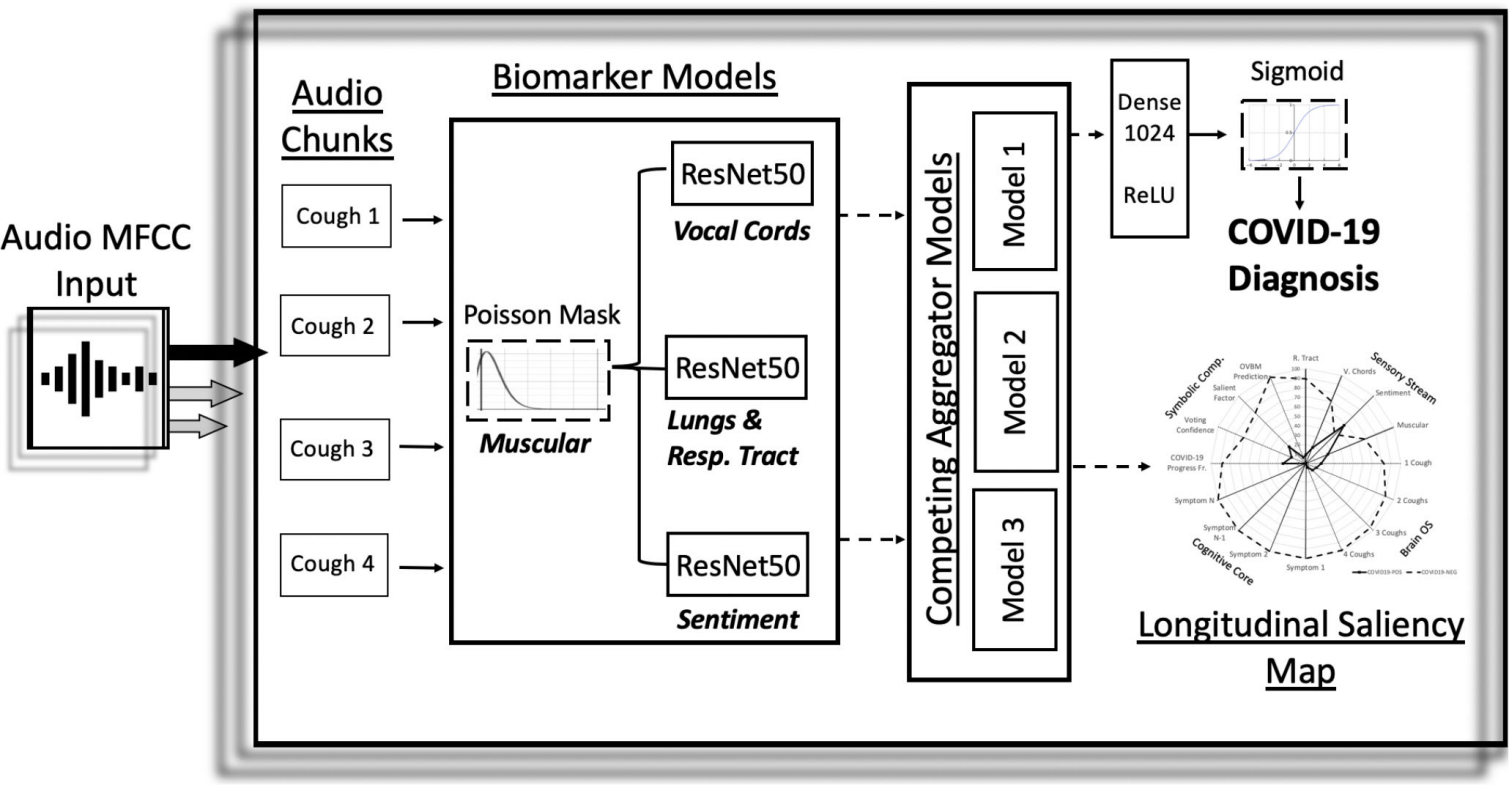
Overview architecture of the COVID-19 discriminator with cough recordings as input, and COVID-19 diagnosis and longitudinal saliency map as output. A similar architecture was used for Alzheimer's [11].

Our proposed architecture, drawn in Fig. 1, takes a recording with one or more coughs, performs two pre-processing steps with the recording and inputs it into a CNN based model to output a pre-screening diagnostic along with a biomarker saliency map (e.g. in Fig. 3(c)).

As pre-processing, each input cough recording is split into 6 second audio chunks, padded as needed, processed with the MFCC package [16] and subsequently passed through biomarker 1. The output of these steps becomes the input to a CNN as described in the next paragraph.

The CNN architecture is made up of three ResNet50s in parallel. The 7 × 7 × 2048 4-d tensor output layer of each ResNet50 model is concatenated in parallel as depicted in Fig. 1. In the baseline models, these ResNet50s are not pre-trained. In the best performing model, they are pre-trained to capture acoustic features on biomarkers 2,3 and 4 as described in Section II.C. The output of these three concatenated tensors is then pooled together using a Global Average Pooling 2D layer, followed by a 1024 neuron deeply connected neural network layer (dense) with ReLU activation, and finally a binary dense layer with sigmoid activation. The whole architecture is trained on the COVID-19 cough dataset for binary classification. The various chunk outputs from the CNN architecture are aggregated using competing schemes to generate the subject's saliency map as illustrated in Fig. 3(c). The results of this paper and presented in Table I are based solely on the first audio chunk outputs. Future work may show that aggregation can not only improve explainability but also increase diagnostic accuracy.

### C. COVID-19 Model Biomarkers

The MIT Open Voice Medicine architecture uses the same four biomarkers we previously tested for the detection of Alzheimer's which achieved above state-of-the-art accuracy [11]. These four biomarkers inspired by medical community choices [17]–[21] are: muscular degradation, changes in vocal cords, changes in sentiment/mood, and changes in the lungs and respiratory tract.

#### 1) Biomarker 1 (Muscular Degradation)

Following memory decay models from [22], [23] we introduced muscle fatigue and degradation features by modifying input signals for all train and test sets with the Poisson mask in Equation 1. Poisson decay is a commonly occurring distribution in nature [24] which has previously been proposed to model muscular degradation. We find it effective since removing this biomarker roughly doubles the error rate in official predictions. To capture the influence of muscular degradation in individual predictions, we developed a muscular degradation metric based on comparing the output with and without this initial Poisson step. This metric is the normalized ratio of the prediction with and without the mask and it is incorporated in the saliency map as illustrated in Fig. 3. For COVID negatives this metric is plotted directly; and for positives we plot one minus this metric.

The Poisson mask applied on a cough recording MFCC point, I_x_, is calculated by multiplying this value by a random Poisson distribution of parameters I_x_ and λ, where λ is the average of all values in the MFCC.

\begin{align*}
&M\left({{I_x}} \right) = Poiss\left(\lambda \right){I_x}\tag{1}\\
&Poiss\left({X = k} \right) = \ \frac{{{\lambda ^k}{e^{ - k}}}}{{k!}}\tag{2}
\end{align*}

#### 2) Biomarker 2 (Vocal cords)

Subjects with lung diseases often have distinct expressions of vocal cords biomarkers as compared to healthy ones [25]. For example, studies have reported phonation threshold pressure, the minimal lung pressure necessary to start and hold vocal fold oscillation, correlates to vocal fatigue [26]. Therefore, we were interested in creating a vocal cord biomarker model capable of detecting changes in basic features of vocal cord sounds in continuous speech.

We focused on developing a Wake Word model [27] for a very universal sound ”mmmmmm”. We trained a ResNet50 [28] with input shape (300, 200) from MFCC to discriminate the word ’Them’ from others using LibriSpeech, an audiobook dataset with ≈1,000 hours of speech [29]. The model was trained by creating a balanced sample set of 11,000 two-second audio chunks, half containing the word and half without it, and achieved a validation accuracy of 89%.

We found that the learned features from this biomarker enable the detection of variations in the vocal cords that exist between COVID-19 and control subjects, discriminating 54% of the test set. As shown in Table II, for 19% of the subjects, this is the only biomarker to correctly discriminate them.

**TABLE II:** The First Three Rows Shows the Unique Percentage of Samples Detected by Each Individual Biomarker for COVID-19 and Alzheimer's, Demonstrating the Discriminatory Value of the Exact Same Three Biomarkers for Both Diseases. The Next Three Rows Focus on the Overlap Between Two Biomarkers Demonstrating How Orthogonal They Are to Each Other

**Biomarker**	**Model Name**	**COVID(%)**	**Alzheimer(%)**
R. Tract	Cough	23	9
Sentiment	Intonation	8	19
Vocal cords	WW 'THEM'	19	16
R.Tract&Sent.	Cough&Tone.	0	0
R.Tract&V.cords	Cough&WW	1	6
Sent.&V.cords	Tone.&WW	0	3
In All 3		34	41
In Neither 3		15	6

#### 3) Biomarker 3 (Sentiment)

Studies [14] show a cognitive decline in COVID-19 patients and clinical evidence supports the importance of sentiments in the early-diagnosis of neurodegenerative decline [19], [30]. Different clinical settings emphasize different sentiments, such as doubt [31] or frustration [31] as possible neurodegenerative indicators. To obtain a biomarker that detects this decline, we trained a Sentiment Speech classifier model to learn sentiment features on the RAVDESS speech dataset [32], which includes actors intonating in 8 emotional states: neutral, calm, happy, sad, angry, fearful, disgust, and surprised. A ResNet50 [28] was trained on 3 second samples for categorical classification of the 8 intonations with input shape (300, 200) from MFCC which achieved 71% validation accuracy.

#### 4) Biomarker 4 (Lungs and Respiratory Tract)

The human cough has already been demonstrated to be helpful in diagnosing several diseases using automated audio recognition [33], [34]. The physical structure of the lungs and respiratory tract get altered with respiratory infections, and in the early days of the COVID-19, epidemiologists listened to the lungs while patients forced coughs as part of their diagnostic methods. There is evidence that many other diseases may be diagnosed using AI on forced-coughs. An algorithm presented by [35] uses audio recognition to analyse coughs for the automated diagnosis of Pertussis - a contagious respiratory disease that if left untreated can be fatal. Algorithms based on cough sounds collected using smartphone devices are already diagnosing pneumonia, asthma and other diseases with high levels of accuracy [36]–[39]. Therefore, a biomarker model capable of capturing features on the lungs and respiratory tract was selected.

Past models we created with a superset of the cough dataset collected through MIT Open Voice for COVID-19 detection [10] accurately predicted a person's gender and mother tongue based on one cough. We hypothesized that such models capable of learning features and acoustic variations on forced coughs trained to differentiate mother tongue could enhance COVID-19 detection using transfer learning. We stripped from the dataset all metadata but the spoken language of the person coughing (English, Spanish), and split audios into 6s chunks. A ResNet50 [28] was trained on binary classification of English vs Spanish with input shape (600, 200) from MFCC and 86% accuracy. We found that the cough biomarker is the one that provides the most relevant features with 23% unique detection and 58% overall detection as shown in Table II.

## III. Results

### A. COVID-19 Forced-Cough Discrimination Accuracy

**Fig. 2.**
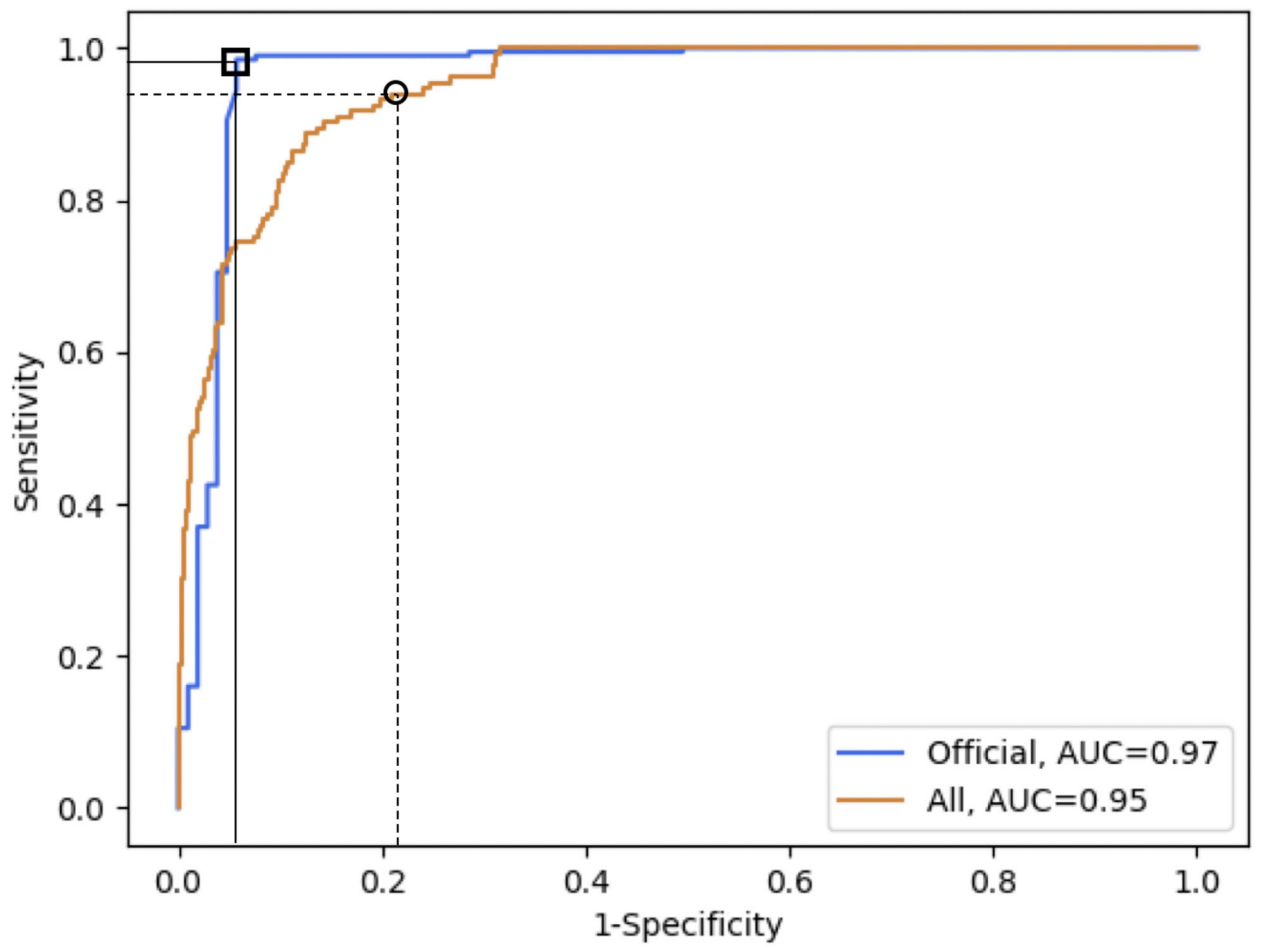
The top orange line with a square shows the ROC curve for the set of subjects diagnosed with an official test with AUC (0.97), while the bottom blue curve with a circle shows the ROC curve for all subjects in the validation set. The square shows the chosen threshold with 98.5% sensitivity and 94.2% specificity on officially tested subjects, and the black circle shows the chosen threshold for high sensitivity (94.0%) on the whole validation set, although any point on the curve could be chosen depending on the use case.

Our model achieves a 97.1% discrimination accuracy on subjects diagnosed with an official test. The fact that our model discriminates officially tested subjects 18% better than self-diagnosed, as shown in Table I, is consistent with this discrepancy being caused by self-diagnostic errors. These errors can contribute to the expansion of the virus even if subjects are well intentioned, and our tool could help diminish this impact. To that end, it is remarkable that our tool discriminates 100% of asymptomatics at the expense of a false positive rate of 16.8%. Note the tool sensitivity/specificity can be tailored depending on the use case, such as improving specificity at the cost of sensitivity, as shown in Fig. 2.

### B. Biomarker Saliency Evaluation

**Fig. 3.**
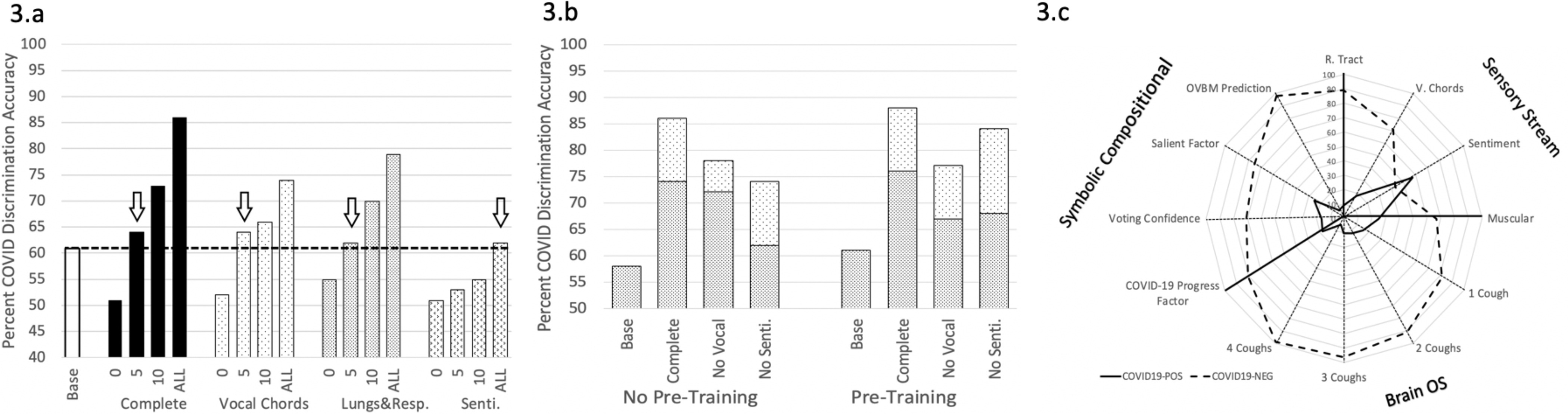
A. The numbers on the x-axis describe the number of layers in the biomarker models fine-tuned to COVID-19. The fewer required to beat the baseline (which is the same architecture trained on COVID-19 discrimination without the pre-trained biomarker models) shows the relevance of each biomarker for COVID-19. “Complete: shows the final COVID-19 discriminator with all the biomarkers integrated. B. The white dotted part of the bar shows the performance gained when the Cough biomarker model is incorporated, while pre-trained denotes individually training the biomarker models for COVID-19 before integrating them into the multi-modal architecture on Fig. 1. C. shows the explainable saliency map derived from biomarker model predictions to longitudinally track patient progression and is analogous to the saliency map derived for Alzheimer's [11]. OVBM denotes the final model diagnostic. The *BrainOS* section shows the model aggregated prediction for 1-4 coughs of a subject. The *COVID-19 progress factor* calculates based on the 1-4 cough predictions, a possible degree of severity from the quantity of acoustic information required for a confident diagnostic. The *voting confidence* and *salient factor* indicate, based on the composite predictions of individual biomarker models, the aggregate confidence and salient discrimination for each subject.

To measure the role of each biomarker in the discrimination task, we compared the results between a baseline model and the complete model with and without each biomarker. The baseline model is defined as the same architecture shown in Fig. 1 trained on COVID-19 discrimination as in our model but without the pre-trained biomarker model features. Therefore, the baseline model has the exact same number of neurons and weights but is initialized randomly instead of with pre-trained models. From Fig. 3(a), the lungs and respiratory track biomarker model requires very few layers to be fine-tuned to COVID-19 discrimination to beat the baseline which emphasizes the relevance of its pre-learned features. Meanwhile, sentiment requires retraining many more features in order to surpass the baseline showing that although the pre-learned features bring value, they may be less closely related. Fig. 3(b) shows leave-one-out significance by measuring the performance loss when a chosen biomarker model is removed. Compared to the sentiment biomarker, the vocal cord biomarker contributes twice the significance in terms of detection accuracy.

We illustrate similar metrics in Table II by showing the percentage of unique patients captured by each biomarker. This is consistent with each biomarker model bringing complementary sets of features, and suggests incorporating additional biomarker models may increase the diagnostic accuracy and explainability of the MIT OVBM AI architecture for COVID-19. Note that the three biomarkers are quite distinct because in pairs they find no unique subjects.

As shown in Fig. 3(c), on top of the diagnostic accuracy for COVID-19, our AI biomarker architecture outputs a set of explainable insights for doctors to analyse the make-up of each individual diagnostics as follows: the Sensory Stream indicates the expression of the chosen biomarkers; the BrainOS shows the model confidence improvement as more coughs from one subject are fed into it, signalling the strength of the diagnosis and in turn potentially of the disease severity; the Symbolic Compositional Models provides a set of composite metrics based on the Sensory Stream and BrainOS. Together, these modular metrics could enable patients to be longitudinally monitored using the saliency map of Fig. 3(c), as well as for the research community to hypothesize new biomarkers and relevant metrics. Future research may demonstrate to what extent our model can promptly detect when a COVID-19 positive subject no longer has the disease and/or is not contagious.

## IV. Discussion

We have proven COVID-19 can be discriminated with 98.5% accuracy using only a forced-cough and an AI biomarker focused approach that also creates an explainable diagnostic in the form of a disease progression saliency chart. We find most remarkable that our model detected all of the COVID-19 positive asymptomatic patients, 100% of them, a finding consistent with other approaches eliciting the diagnostic value of speech [40].

Our research uncovers a striking similarity between Alzheimer's and COVID discrimination. The exact same biomarkers can be used as a discrimination tool for both, suggesting that perhaps, in addition to temperature, pressure or pulse, there are some higher-level biomarkers that can sufficiently diagnose conditions across specialties once thought mostly disconnected. This supports shared approaches to data collection as suggested by the MIT Open Voice team [27].

This first stage of developing the model focused on training it on a large dataset to learn good features for discriminating COVID-19 forced-coughs. Although coughs from subjects that were diagnosed through personal or doctor assessment might not be 100% correctly labelled, they enable training the model on a significant variety and quantity of data, essential to reduce bias and improve model robustness. Thus, we feel the results on the set of subjects diagnosed with an official test serve as an indicator that the model would have similar accuracy when deployed, and to verify this we are now undergoing clinical trials in multiple hospitals. We will also gather more quality data that can further train, fine-tune, and validate the model.

Since there are cultural and age differences in coughs, future work could also focus on tailoring the model to different age groups and regions of the world using the metadata captured, and possibly including other sounds or input modalities such as vision or natural language symptom descriptions.

Another issue that may be researched is whether cough segmentation can improve the results. For the screening outputs to have diagnostic validity, there must be a process to verify recordings correspond to coughs. In the official tests of our dataset only three recordings corresponded to speech instead of coughs and we had to sort these manually since there is still no way to do so automatically.

This non-invasive, free, real-time pre-screening tool may prove to have a great potential to complement current efforts to contain the disease in low-infected areas as well as to mitigate the impact in highly-infected areas, where unconscious asymptomatics may spread the virus. We contend the MIT Open Voice approach presented has great potential to work in parallel with healthcare systems to augment current approaches to manage the spread of the pandemic, especially if combined with broader uses of an open approach, as is being attempted by the https://www.openvoicenetwork.org. We present some possible example use cases:

*Population daily screening tool:* As workers go back to work, students go back to school, and commuters use public transport, to name a few, methods are required to screen infected COVID-19 carriers, especially asymptomatics. The only screening method currently available is using thermometers, however this study [41] showed only 45% of mild-moderate COVID-19 cases have fever (this represents 9% of COVID-19 positives when asymptomatics are included). Meanwhile our tool detects 98.5% of COVID-19 positives, including 100% of asymptomatics.

**Fig. 4.**
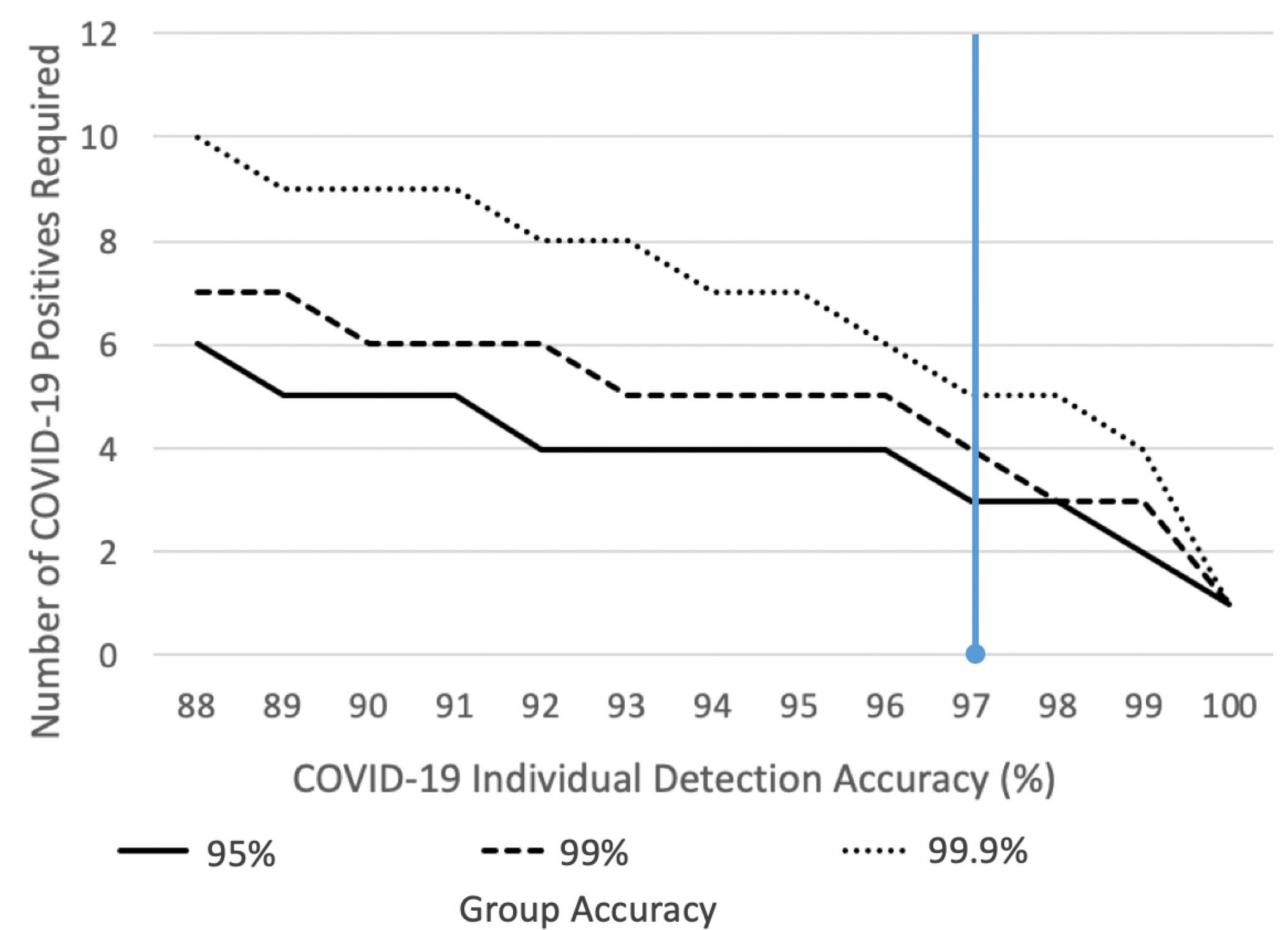
In cases where there are very few infected individuals, a group pre-screening tool can be derived from the COVID-19 OVBM model to accurately alert infected groups while avoiding false-positives as illustrated in the graph. With the current accuracy, shown in blue, a threshold of 3 positives in a group of 25 are required so that only 1% of groups of 25 with no cases are falsely labelled and therefore unnecessarily tested via expensive biological tests. In other words, in a campus with 2500 yet uninfected students, only 25 will have to be tested with biological methods until 3 people in a class of 25 catch the virus, in which case the screening will alert of the outbreak. The x-axis shows how the required number of positives in a group, 3 in this example, drops if the COVID-19 model accuracy improves. Each line shows percent of groups of 25 people falsely tagged with COVID-19 with a minimum number of COVID-19 positives in it. As a second example, assume a country like New Zealand, with very few COVID-19 cases, wanted to screen for new early outbreaks and to do so tested 50M inhabitants using a PCR or serology test with 99% specificity. The country would purchase 50M tests and obtain 500 000 false-positives. Meanwhile, assume a group test yielding a 99.9% test accuracy was used, i.e. requiring 5 positives instead of 3 in the example above. Of the, 2M groups of 25, only 2000 groups would be falsely tagged or 50 000 people. Hence, 0.1% of the cost and 0.1% of the false positives otherwise. The value of this group testing tool is that it enables organizations and countries to pre-screen its whole population daily, and rapidly locate incipiently infected groups, without the necessity of using an expensive PCR or serology test on each inhabitant.

*Pre-selection of candidates for test pooling:* The test pooling strategy is expected to be employed in many countries, especially in low-incidence areas to rapidly identify a sub group of individuals likely to be infected, however, “preliminary results show there is no dilution and no decrease on test sensitivity when minipools of five samples each are used” [42]. Group testing with our tool as shown in Fig. 4, could pre-screen school classrooms, factories or even countries on a daily basis signalling probable infected candidate groups for smaller test pooling batches.

*COVID-19 test in countries where PCR/serology testing is not possible:* The availability of COVID-19 tests worldwide is far from evenly distributed. “Even where there is enough money, many African health authorities are unable to obtain the supplies needed as geopolitically powerful countries mobilise economic, political, and strategic power to procure stocks for their populations” [43]. This pre-screening tool has the potential to bring large-scale detection to areas of the world were testing is too expensive or logistically complex, essential to halt the spread of the disease worldwide.

## V. Conclusion

We have created an AI pre-screening test that discriminates 98.5% COVID-19 positives from a forced-cough recording, including 100% of asymptomatics, at essentially no cost and with an accompanying saliency map for longitudinal explainability.

A group outbreak detection tool could be derived from this model to pre-screen whole-populations on a daily basis, while avoiding the cost of testing each inhabitant, especially important in low-incidence areas where the required post-test confinement is harder to justify. Figure 4 shows that by deriving the COVID-19 cough discrimination model for a group test, it can correctly detect the presence of COVID-19 in 99.9% of groups of 25 people with 5 positives, and 95% of groups with 3 positives.

As part of our ongoing clinical trials, data pipelines with hospitals worldwide have been setup to continue to improve the tool including: Mount Sinai and White Planes Hospitals in the US, Catalan Health Institute in Catalonia, Hospitales Civiles de Guadalajara in Mexico, and Ospedale Luigi Sacco in Italy. We plan on leveraging this data to further train and validate our models with the aim of improving pandemic management practices. Note from Fig. 4 how the number of COVID-19 positives required in group testing greatly drops as the individual model improves, calling for a larger database and further refinement of our model.

To that end, we have reached an agreement with a Fortune 100 company to demonstrate the value of our tool as part of their COVID-19 management practices. As we have shown there are cultural and age differences in coughs, future work could focus on tailoring the model to different age groups and regions of the world using the metadata captured, something we would like to test at the company site.

Eventually we hope our research methods inspire others to develop similar and complementary approaches to disease management beyond dementia and COVID-19, possibly expanding our initial set of orthogonal audio biomarkers. We have followed the MIT Open Voice approach [27], [10] that postulates voice samples may eventually be broadly available if shared by smart speakers and other ever-listening devices such as your phone. Voice may be combined into a multi-modal approach including vision, EEG and other sensors. Pandemics could be a thing of the past if pre-screening tools are always-on in the background and constantly improved. In [44] we introduce “Wake Neutrality” as a possible approach to make that vision a reality while we discuss associated legal hurdles.
